# Synthesis and structure of 5,5′-(tris­ulfane-1,3-di­yl)bis­(1,3,4-thia­diazol-2-amine)

**DOI:** 10.1107/S2056989026005517

**Published:** 2026-06-05

**Authors:** Aziz Atashov, Batirbay Torambetov, Jamshid Ashurov, Tuncer Hökelek, Alebel N. Belay, Asmet N. Azizova

**Affiliations:** a Karakalpak State University, 1 Ch. Abdirov St., Nukus 230112, Uzbekistan; b National University of Uzbekistan named after Mirzo Ulugbek 4 University St Tashkent 100174 Uzbekistan; c Institute of Bioorganic Chemistry, Academy of Sciences of Uzbekistan, M. Ulugbek St. 83, Tashkent 100125, Uzbekistan; d Hacettepe University, Department of Physics, 06800 Beytepe-Ankara, Türkiye; e Department of Chemistry, Bahir Dar University, PO Box 79, Bahir Dar, Ethiopia; f Azerbaijan Medical University, Scientific Research Centre (SRC), A. Kasumzade Str. 14, AZ 1022, Baku, Azerbaijan; g Scientific Research Center, Baku Engineering University, Hasan Aliyev Str. 120, AZ 0101, Khirdalan, Absheron, Azerbaijan; University of Aberdeen, United Kingdom

**Keywords:** 1,3,4-thia­diazole, crystal structure, noncovalent inter­actions

## Abstract

The title compound, C_4_H_4_N_6_S_5_, consists of two 1,3,4-thia­diazol-2-amine moieties bridged by a tris­ulfanediyl group [S—S—S = 107.98 (6)°]. The conformation is supported by an intra­molecular π–π stacking inter­action. In the crystal, N—H⋯N hydrogen bonds link the mol­ecules, enclosing *R*^2^_2_(8) and *R*^5^_5_(31) ring motifs, into infinite channels/tubes propagating along the *b*-axis direction. Hirshfeld surface analysis revealed that the most important contributions for the crystal packing are from S⋯S (33.6%) and H⋯N/N⋯H (32.8%) inter­actions.

## Abstract 2

The title compound contains two 1,3,4-thia­diazol-2-amine moieties bridged by a tris­ulfanediyl group. In the crystal, N—H⋯N hydrogen bonds generate infinite [010] chains.

## 1. Chemical context

1,3,4-Thia­diazole (C_2_H_2_N_2_S) is a five-membered heterocyclic aromatic compound containing two nitro­gen atoms and one sulfur atom. In order to improve the functional properties of 1,3,4- thia­diazo­les, substituents can be attached at the 2- and 5-positions, enabling the creation of diverse bioactive compounds (*e.g.*, anti­bacterial, anti­cancer) from a stable, electron-deficient, five-membered heterocyclic ring (Hu *et al.*, 2014 ▸). Common synthesis methods include the cyclization of thio­semicarbazides or di­acyl­hydrazines, as well as nucleophilic substitution and C—H activation to introduce various substituents (Hu *et al.*, 2014 ▸; Kumar *et al.*, 2024 ▸). In this work, we describe the synthesis and structure of the title compound, C_4_H_4_N_6_S_5_ (**I**), prepared by the oxidation of 5-amino-1,3,4-thia­diazole-2-thiol with 30% H_2_O_2_.
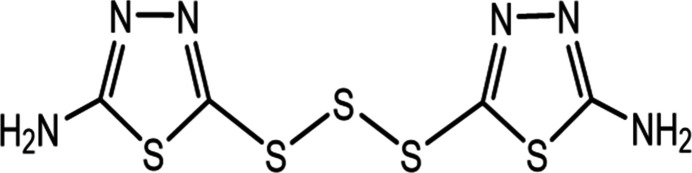


## 2. Structural commentary

**Figure 1:**
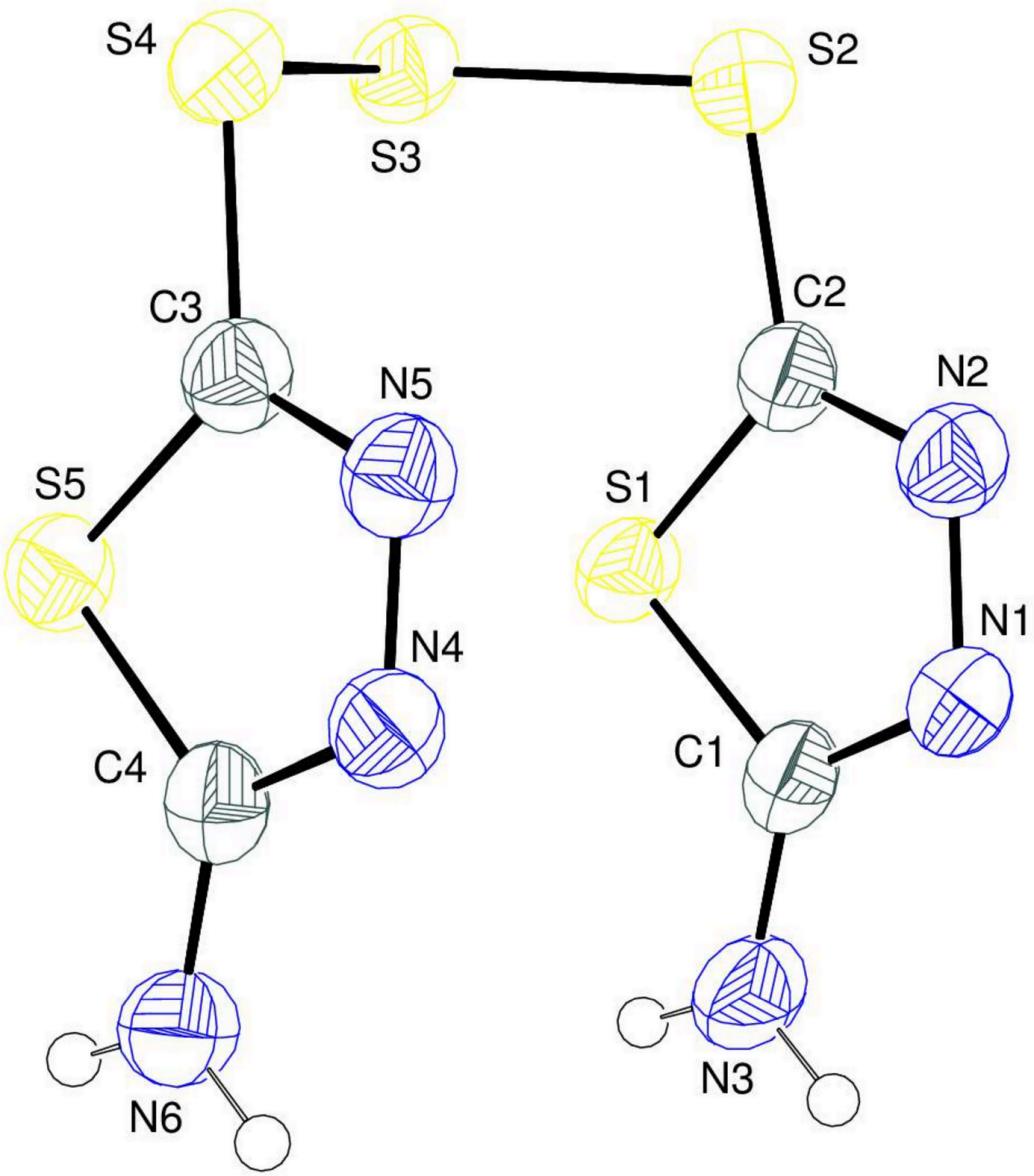
The mol­ecular structure of (**I**) showing 50% probability ellipsoids.

Compound (**I**) consists of two 1,3,4-thia­diazol-2-amine moieties bridged by the tris­ulfanediyl group (Fig. 1 ▸) with the S2—S3—S4 bridging angle of 107.98 (6)°. The S2—S3 [2.0478 (16) Å] and S3—S4 [2.0705 (16) Å] and S2—C2 [1.766 (5) Å] and S4—C3 [1.755 (16) Å] bond lengths are slightly different, while the C2—S2—S3 [101.91 (15)°] and C3—S4—S3 [100.29 (15)°] bond angles are significantly different. The *A* (N1/N2/S1/C1/C2) and *B* (N4/N5/S5/C3/C4) rings are oriented at a dihedral angle of 5.15 (13)°, with a centroid–centroid separation of 3.621 (2) Å (slippage 1.336 Å), indicative of an intra­molecular π–π stacking inter­action. The key torsion angles associated with the tris­ulfide bridge are C2—S2—S3—S4 = 81.8 (3) and S2—S3—S4—C3 = −79.3 (3)°.

Atoms N3, N6, S2 and S4 are displaced by 0.076 (4), −0.062 (4), 0.0182 (11) and −0.1483 (12) Å, respectively, from their corresponding ring planes. The C1—N3 [1.338 (6) Å] and C4—N6 [1.320 (6) Å] bond lengths are a little longer than a typical C=N double bond (*e.g.* 1.27–1.30 Å) in imines and oximes with more orbital overlap indicating partial double bond (*e.g*., 1.35–1.38 Å for pyridine and amides) character due to resonance delocalization. On the other hand, the S1—C1—N3 [120.6 (3)°] and S5—C4—N6 [123.5 (3)°], N1—C1—N3 [125.2 (4)°] and N4—C4—N6 [123.7 (4)°], S1—C1—N1 [114.2 (4)°] and S5—C4—N4 [112.8 (3)°], C1—N1—N2 [111.4 (4)°] and C4—N4—N5 [112.6 (3)°] bond angles are significantly different.

## 3. Supra­molecular features

**Figure 2:**
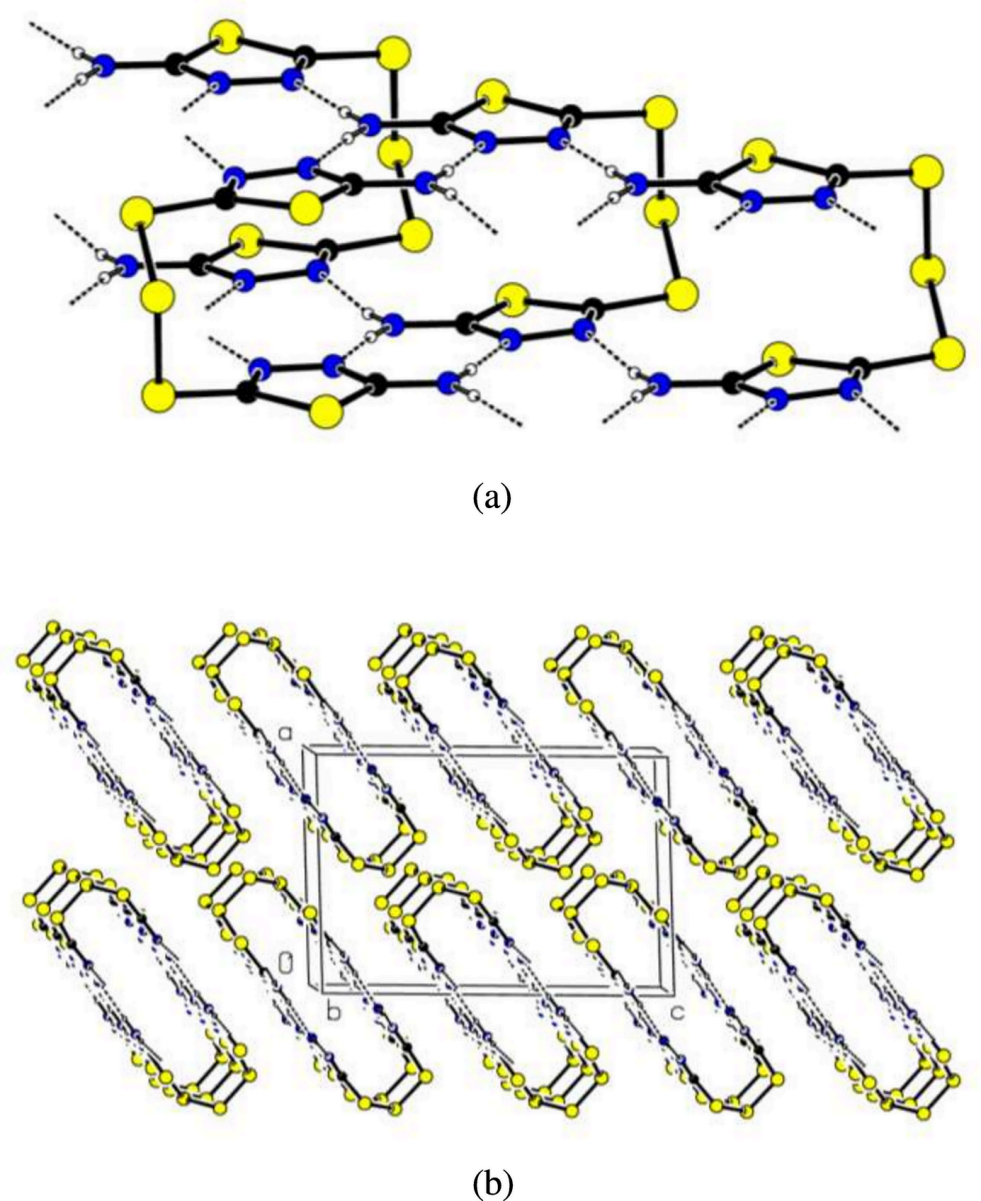
Partial packing diagrams for (**I**) showing N—H⋯N hydrogen bonds as dashed lines with (*a*) the 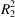
(8) and 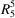
(31) ring motifs and (*b*) the infinite channels/tubes viewed along the *b*-axis direction.

In the crystal, N—H⋯N hydrogen bonds (Table 1 ▸) link the mol­ecules, enclosing 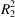
(8) and 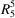
(31) ring motifs (Fig. 2 ▸*a*), into infinite channels/tubes propagating along the *b*-axis direction (Fig. 2 ▸*b*). No inter­molecular π–π stacking or C—H⋯π inter­actions are observed.

## 4. Hirshfeld surface analysis

**Figure 3:**
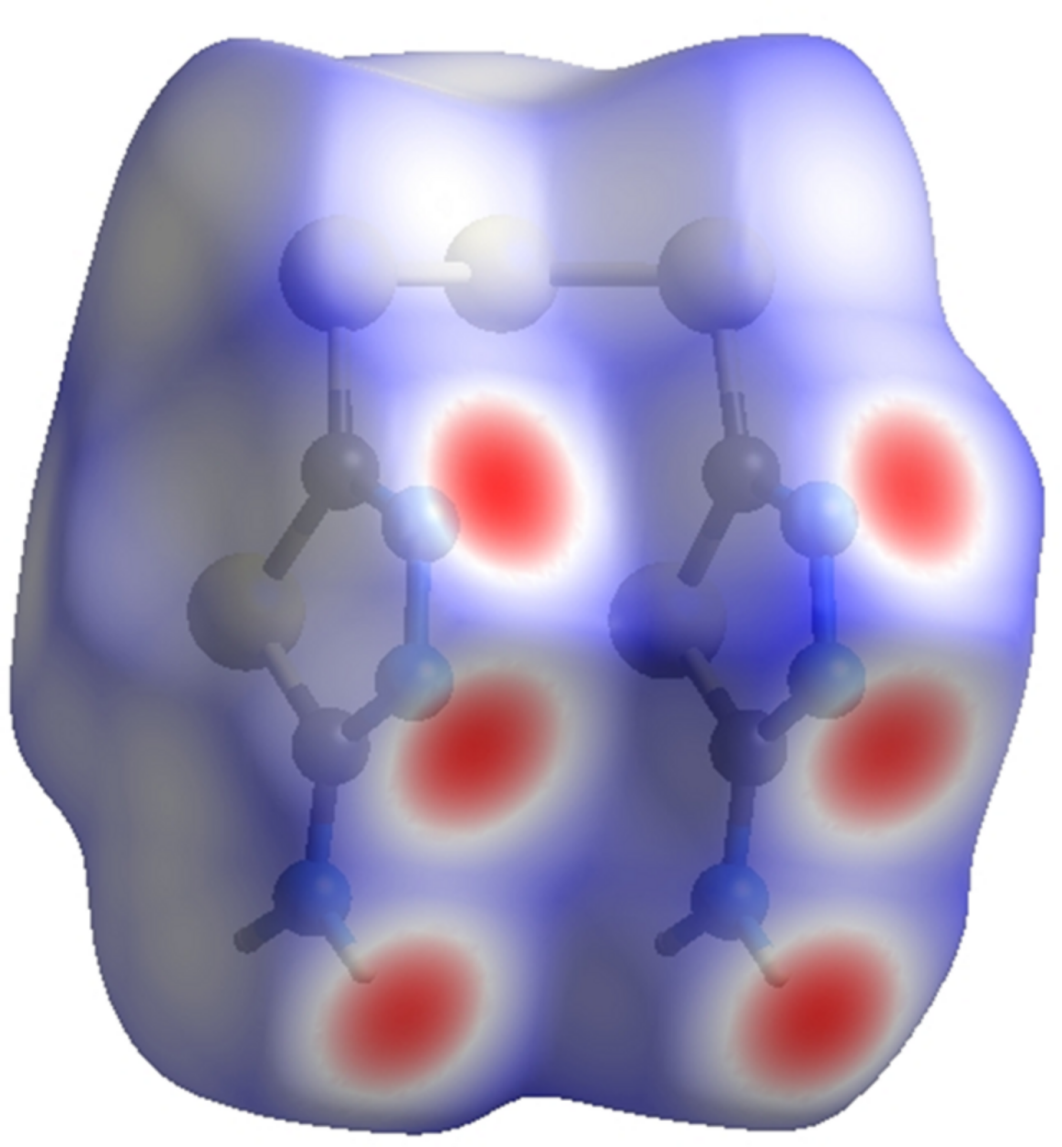
View of the three-dimensional Hirshfeld surface for (**I**) plotted over *d*_norm_ in the range −0.51 to 1.25 a.u.

**Figure 4:**
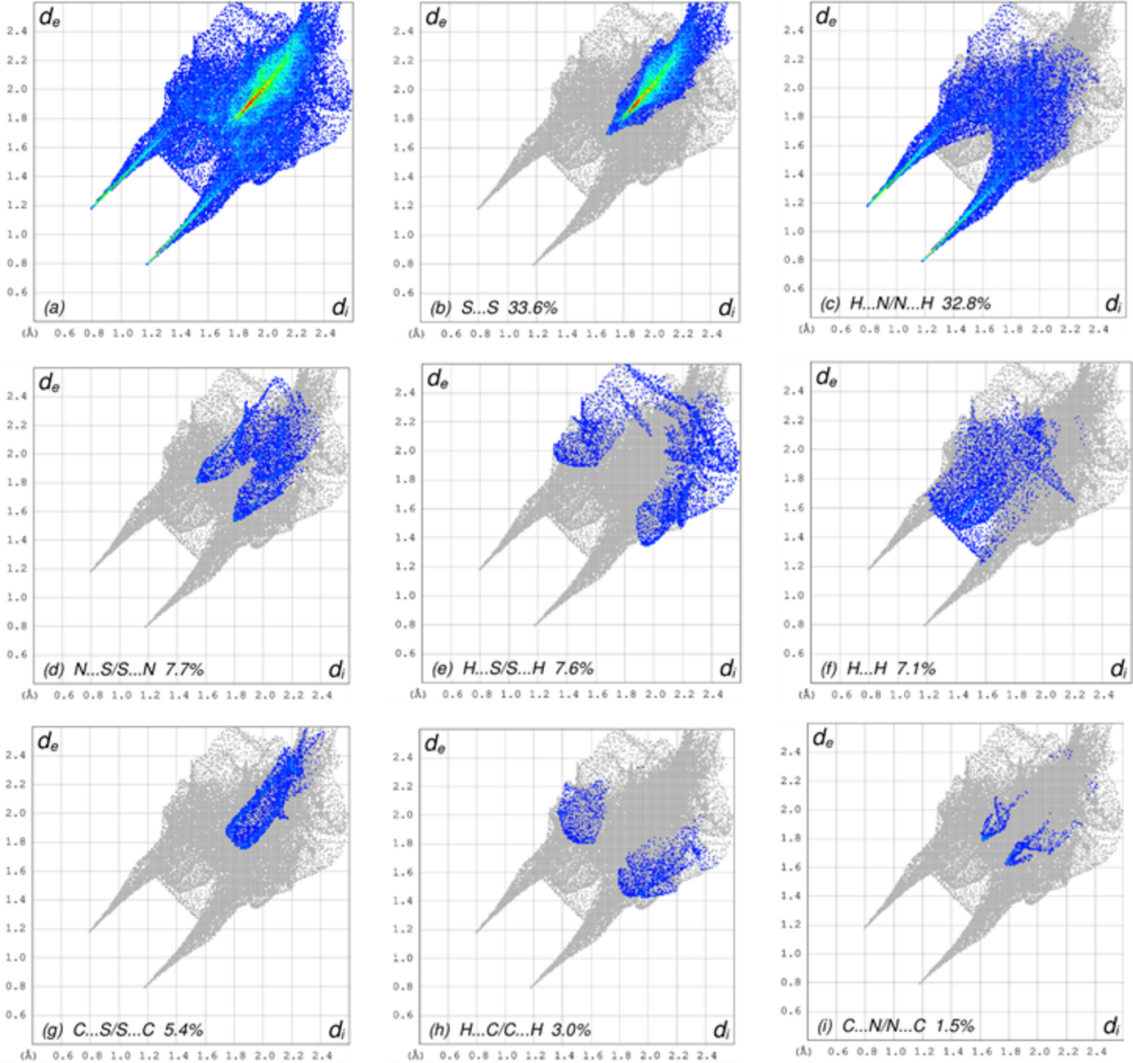
The two-dimensional fingerprint plots for (**I**), showing (*a*) all inter­actions, and delineated into different contact types (*b*)–(*i*). The *d*_i_ and *d*_e_ values are the closest inter­nal and external distances (in Å) from given points on the Hirshfeld surface.

The inter­molecular inter­actions in the crystal were further visualized by carrying out a Hirshfeld surface (HS) analysis using *CrystalExplorer* 17.5 (Spackman *et al.*, 2021 ▸). Fig. 3 ▸ shows the Hirshfeld surface with several neighboring mol­ecules in the crystal. The white surface indicates contacts with distances equal to the sum of van der Waals radii, and the red and blue colours indicate distances shorter (in close contact) or longer (distinct contacts) than the van der Waals radii, respectively. The red spots indicate their roles as the respective donors and/or acceptor atoms in hydrogen bonding, as discussed above; they also appear as the blue and red regions corresponding to positive and negative potentials on the HS mapped over electrostatic potential as shown in Fig. S1. The blue and red regions indicate positive (hydrogen-bond donors) and negative (hydrogen-bond acceptors) electrostatic potentials. The overall two-dimensional fingerprint plots are shown in Fig. 4 ▸*a* and those delineated into different contact types are illustrated in Fig. 4 ▸*b*–*i*. According to the two-dimensional fingerprint plots, S⋯S and H⋯N/N⋯H contacts make the most significant contributions to the HS, at 33.6% and 32.8%, respectively.

## 5. Synthesis and crystallization

**Figure 5:**
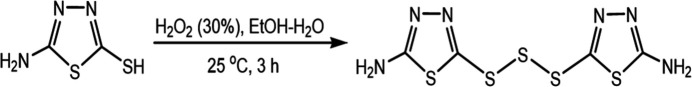
Synthesis scheme for (**I**).

Hydrogen peroxide (30%, 10.4 ml) was added dropwise to a solution of 2-amino-5-mercapto-1,3,4-thia­diazole (0.20 mol) in the mixed solvents of ethanol (40 ml) and water (20 ml) at room temperature (Fig. 5 ▸). The mixture was stirred for 3 h, giving a precipitate. The precipitate was filtered off, dried and recrystallized from a *N*,*N*-di­methyl­formamide (DMF) solution to yield the title compound as a yellow solid. Yellow block-like single crystals of (**I**) suitable for single-crystal X-ray diffraction were grown by slow evaporation from DMF at room temperature. Yield: 58% (based on 2-amino-5-mercapto-1,3,4-thia­diazole). Analysis (%) calculated for C_4_H_4_N_6_S_5_, calculated (observed): C 16.21 (16.18), H 1.36 (1.34), N 28.35 (28.33). IR (ATR, 298 K, cm^−1^): 3123, 3260 and 3402 ν(N—H), 1595 and 1614 ν(C=N). ^1^H NMR (400 MHz, DMSO-*d*_6_, ppm): δ 7.66 and 7.83 (4H, 2 NH_2_). ^13^C{^1^H} NMR (100 MHz, DMSO-*d*_6_, ppm): δ 157.2 and 148.6.

## 6. Refinement

Crystal data, data collection and structure refinement details are summarized in Table 2 ▸. The hydrogen-atom positions were calculated geometrically at distances of N—H = 0.86 Å and refined using a riding model. The constraint *U*_iso_(H) = 1.2*U*_eq_(N) was applied in all cases.

## Supplementary Material

Crystal structure: contains datablock(s) I, global. DOI: 10.1107/S2056989026005517/hb8214sup1.cif

Structure factors: contains datablock(s) I. DOI: 10.1107/S2056989026005517/hb8214Isup2.hkl

Figure S1.3D Hirshfeld surface of the title compound. DOI: 10.1107/S2056989026005517/hb8214sup3.pdf

Supporting information file. DOI: 10.1107/S2056989026005517/hb8214Isup4.cml

CCDC reference: 2556705

Additional supporting information:  crystallographic information; 3D view; checkCIF report

## Figures and Tables

**Table 1:** Hydrogen-bond geometry (Å, °)

*D*—H⋯*A*	*D*—H	H⋯*A*	*D*⋯*A*	*D*—H⋯*A*
N3—H3*A*⋯N4^i^	0.86	2.12	2.969 (6)	171
N3—H3*B*⋯N2^ii^	0.86	2.25	3.099 (6)	170
N6—H6*A*⋯N5^ii^	0.86	2.16	3.021 (5)	174
N6—H6*B*⋯N1^i^	0.86	2.16	3.015 (6)	171

Symmetry codes: (i) 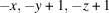; (ii) 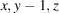.

**Table 2:** Experimental details

Crystal data	
Chemical formula	C_4_H_4_N_6_S_5_
*M*_r_	296.43
Crystal system, space group	Monoclinic, *P*2_1_/*c*
Temperature (K)	293
*a*, *b*, *c* (Å)	11.0300 (4), 5.9139 (2), 16.2881 (7)
β (°)	92.406 (4)
*V* (Å^3^)	1061.54 (7)
*Z*	4
Radiation type	Cu *K*α
μ (mm^−1^)	9.89
Crystal size (mm)	0.16 × 0.12 × 0.08

Data collection	
Diffractometer	XtaLAB Synergy, Single source at home/near, HyPix3000
Absorption correction	Multi-scan (*CrysAlis PRO*; Rigaku OD, 2020 ▸)
*T*_min_, *T*_max_	0.380, 1.000
No. of measured, independent and observed [*I* > 2σ(*I*)] reflections	8905, 2054, 1675
*R*_int_	0.078
(sin θ/λ)_max_ (Å^−1^)	0.615

Refinement	
*R*[*F*^2^ > 2σ(*F*^2^)], *wR*(*F*^2^), *S*	0.069, 0.202, 1.00
No. of reflections	2054
No. of parameters	136
H-atom treatment	H-atom parameters constrained
Δρ_max_, Δρ_min_ (e Å^−3^)	0.77, −0.66

Computer programs: *CrysAlis PRO* (Rigaku OD, 2020 ▸), *SHELXT* (Sheldrick, 2015*a* ▸), *SHELXL2016/6* (Sheldrick, 2015*b* ▸) and *OLEX2* (Dolomanov *et al.*, 2009 ▸).

## supplementary crystallographic information

### 5,5'-(Trisulfane-1,3-diyl)bis(1,3,4-thiadiazol-2-amine) Crystal data

**Table:**

C_4_H_4_N_6_S_5_	*F*(000) = 600
*M**_r_* = 296.43	*D*_x_ = 1.855 Mg m^−^^3^
Monoclinic, *P*2_1_/*c*	Cu *K*α radiation, λ = 1.54184 Å
*a* = 11.0300 (4) Å	Cell parameters from 3057 reflections
*b* = 5.9139 (2) Å	θ = 4.0–71.0°
*c* = 16.2881 (7) Å	µ = 9.89 mm^−^^1^
β = 92.406 (4)°	*T* = 293 K
*V* = 1061.54 (7) Å^3^	Block, colourless
*Z* = 4	0.16 × 0.12 × 0.08 mm

### 5,5'-(Trisulfane-1,3-diyl)bis(1,3,4-thiadiazol-2-amine) Data collection

**Table:**

XtaLAB Synergy, Single source at home/near, HyPix3000 diffractometer	2054 independent reflections
Radiation source: micro-focus sealed X-ray tube, PhotonJet (Cu) X-ray Source	1675 reflections with *I* > 2σ(*I*)
Mirror monochromator	*R*_int_ = 0.078
Detector resolution: 10.0000 pixels mm^-1^	θ_max_ = 71.5°, θ_min_ = 4.0°
ω scans	*h* = −13→13
Absorption correction: multi-scan (CrysAlisPro; Rigaku OD, 2020)	*k* = −7→7
*T*_min_ = 0.380, *T*_max_ = 1.000	*l* = −19→19
8905 measured reflections	

### 5,5'-(Trisulfane-1,3-diyl)bis(1,3,4-thiadiazol-2-amine) Refinement

**Table:**

Refinement on *F*^2^	Primary atom site location: dual
Least-squares matrix: full	Hydrogen site location: inferred from neighbouring sites
*R*[*F*^2^ > 2σ(*F*^2^)] = 0.069	H-atom parameters constrained
*wR*(*F*^2^) = 0.202	*w* = 1/[σ^2^(*F*_o_^2^) + (0.1567*P*)^2^] where *P* = (*F*_o_^2^ + 2*F*_c_^2^)/3
*S* = 1.00	(Δ/σ)_max_ < 0.001
2054 reflections	Δρ_max_ = 0.77 e Å^−^^3^
136 parameters	Δρ_min_ = −0.66 e Å^−^^3^
0 restraints	

### 5,5'-(Trisulfane-1,3-diyl)bis(1,3,4-thiadiazol-2-amine) Special details

**Table:**

Geometry. All esds (except the esd in the dihedral angle between two l.s. planes) are estimated using the full covariance matrix. The cell esds are taken into account individually in the estimation of esds in distances, angles and torsion angles; correlations between esds in cell parameters are only used when they are defined by crystal symmetry. An approximate (isotropic) treatment of cell esds is used for estimating esds involving l.s. planes.

### 5,5'-(Trisulfane-1,3-diyl)bis(1,3,4-thiadiazol-2-amine) Fractional atomic coordinates and isotropic or equivalent isotropic displacement parameters (Å^2^)

**Table:**

	*x*	*y*	*z*	*U*_iso_*/*U*_eq_	
S3	0.47315 (9)	0.7178 (2)	0.31102 (7)	0.0452 (4)	
S5	0.21531 (10)	0.30438 (19)	0.26530 (7)	0.0457 (4)	
S1	0.33652 (10)	0.49558 (19)	0.47085 (7)	0.0451 (4)	
S4	0.33425 (11)	0.75476 (18)	0.22253 (7)	0.0464 (4)	
S2	0.44180 (10)	0.93820 (19)	0.40475 (7)	0.0488 (4)	
N2	0.2520 (3)	0.8882 (7)	0.5007 (2)	0.0466 (9)	
N4	0.0612 (3)	0.5350 (6)	0.3441 (2)	0.0438 (8)	
N5	0.1361 (3)	0.6891 (6)	0.3097 (2)	0.0444 (8)	
N1	0.1785 (4)	0.7401 (7)	0.5423 (3)	0.0473 (9)	
N6	0.0369 (4)	0.1443 (6)	0.3578 (3)	0.0489 (9)	
H6B	−0.023827	0.161979	0.388611	0.059*	
H6A	0.061155	0.010410	0.345985	0.059*	
N3	0.1525 (4)	0.3479 (7)	0.5594 (3)	0.0549 (11)	
H3A	0.090114	0.365799	0.588721	0.066*	
H3B	0.178061	0.214081	0.548935	0.066*	
C4	0.0930 (4)	0.3226 (7)	0.3287 (3)	0.0409 (9)	
C1	0.2097 (4)	0.5284 (8)	0.5301 (3)	0.0431 (10)	
C2	0.3355 (4)	0.7875 (8)	0.4616 (3)	0.0417 (9)	
C3	0.2197 (4)	0.5970 (8)	0.2674 (3)	0.0417 (9)	

### 5,5'-(Trisulfane-1,3-diyl)bis(1,3,4-thiadiazol-2-amine) Atomic displacement parameters (Å^2^)

**Table:**

	*U*^11^	*U*^22^	*U*^33^	*U*^12^	*U*^13^	*U*^23^
S3	0.0374 (6)	0.0526 (7)	0.0465 (7)	0.0010 (4)	0.0108 (5)	−0.0029 (5)
S5	0.0428 (6)	0.0443 (6)	0.0507 (7)	0.0035 (4)	0.0097 (5)	−0.0090 (5)
S1	0.0425 (6)	0.0455 (6)	0.0481 (7)	0.0052 (4)	0.0121 (5)	−0.0021 (4)
S4	0.0486 (7)	0.0500 (7)	0.0408 (7)	−0.0005 (4)	0.0061 (5)	0.0013 (5)
S2	0.0502 (7)	0.0490 (7)	0.0479 (7)	−0.0088 (5)	0.0100 (5)	−0.0070 (5)
N2	0.0414 (19)	0.051 (2)	0.048 (2)	0.0010 (16)	0.0068 (16)	−0.0047 (18)
N4	0.0376 (17)	0.0445 (19)	0.050 (2)	0.0026 (15)	0.0063 (15)	−0.0057 (16)
N5	0.0423 (18)	0.049 (2)	0.042 (2)	0.0024 (16)	0.0005 (15)	−0.0024 (16)
N1	0.047 (2)	0.049 (2)	0.047 (2)	−0.0013 (16)	0.0141 (17)	−0.0069 (16)
N6	0.0481 (19)	0.042 (2)	0.057 (2)	0.0034 (16)	0.0115 (17)	−0.0026 (17)
N3	0.062 (2)	0.047 (2)	0.058 (3)	0.0075 (19)	0.023 (2)	−0.0003 (18)
C4	0.0351 (19)	0.047 (2)	0.041 (2)	0.0045 (16)	−0.0028 (17)	−0.0067 (18)
C1	0.043 (2)	0.050 (2)	0.037 (2)	0.0039 (18)	0.0058 (17)	−0.0066 (18)
C2	0.040 (2)	0.046 (2)	0.039 (2)	0.0009 (17)	0.0032 (17)	−0.0053 (18)
C3	0.039 (2)	0.044 (2)	0.043 (2)	0.0025 (17)	0.0041 (17)	−0.0028 (18)

### 5,5'-(Trisulfane-1,3-diyl)bis(1,3,4-thiadiazol-2-amine) Geometric parameters (Å, º)

**Table:**

S3—S4	2.0705 (16)	N4—N5	1.366 (5)
S3—S2	2.0478 (16)	N4—C4	1.331 (5)
S5—C4	1.737 (4)	N5—C3	1.294 (6)
S5—C3	1.732 (5)	N1—C1	1.315 (6)
S1—C1	1.744 (4)	N6—H6B	0.8600
S1—C2	1.733 (5)	N6—H6A	0.8600
S4—C3	1.755 (4)	N6—C4	1.320 (6)
S2—C2	1.766 (5)	N3—H3A	0.8600
N2—N1	1.389 (5)	N3—H3B	0.8600
N2—C2	1.287 (6)	N3—C1	1.338 (6)
			
S2—S3—S4	107.98 (6)	C1—N3—H3B	120.0
C3—S5—C4	87.0 (2)	N4—C4—S5	112.8 (3)
C2—S1—C1	86.2 (2)	N6—C4—S5	123.5 (3)
C3—S4—S3	100.29 (15)	N6—C4—N4	123.7 (4)
C2—S2—S3	101.91 (15)	N1—C1—S1	114.2 (4)
C2—N2—N1	113.2 (4)	N1—C1—N3	125.2 (4)
C4—N4—N5	112.6 (3)	N3—C1—S1	120.6 (3)
C3—N5—N4	113.2 (4)	S1—C2—S2	123.1 (3)
C1—N1—N2	111.4 (4)	N2—C2—S1	114.8 (4)
H6B—N6—H6A	120.0	N2—C2—S2	122.0 (4)
C4—N6—H6B	120.0	S5—C3—S4	122.9 (2)
C4—N6—H6A	120.0	N5—C3—S5	114.3 (3)
H3A—N3—H3B	120.0	N5—C3—S4	122.7 (4)
C1—N3—H3A	120.0		
			
S3—S4—C3—S5	−78.2 (3)	N1—N2—C2—S2	−179.3 (3)
S3—S4—C3—N5	97.0 (4)	C4—S5—C3—S4	174.1 (3)
S3—S2—C2—S1	33.6 (3)	C4—S5—C3—N5	−1.4 (3)
S3—S2—C2—N2	−147.6 (4)	C4—N4—N5—C3	1.9 (6)
N2—N1—C1—S1	3.1 (5)	C1—S1—C2—S2	−179.4 (3)
N2—N1—C1—N3	−177.1 (4)	C1—S1—C2—N2	1.8 (4)
N4—N5—C3—S5	0.0 (5)	C2—S1—C1—N1	−2.7 (4)
N4—N5—C3—S4	−175.5 (3)	C2—S1—C1—N3	177.4 (4)
N5—N4—C4—S5	−3.0 (5)	C2—N2—N1—C1	−1.7 (6)
N5—N4—C4—N6	177.6 (4)	C3—S5—C4—N4	2.5 (3)
N1—N2—C2—S1	−0.4 (5)	C3—S5—C4—N6	−178.1 (4)

### 5,5'-(Trisulfane-1,3-diyl)bis(1,3,4-thiadiazol-2-amine) Hydrogen-bond geometry (Å, º)

**Table:**

*D*—H···*A*	*D*—H	H···*A*	*D*···*A*	*D*—H···*A*
N3—H3*A*···N4^i^	0.86	2.12	2.969 (6)	171
N3—H3*B*···N2^ii^	0.86	2.25	3.099 (6)	170
N6—H6*A*···N5^ii^	0.86	2.16	3.021 (5)	174
N6—H6*B*···N1^i^	0.86	2.16	3.015 (6)	171

Symmetry codes: (i) −*x*, −*y*+1, −*z*+1; (ii) *x*, *y*−1, *z*.
