# Supplementary material for: Synthesis and structure of 5,5′-(tris­ulfane-1,3-di­yl)bis­(1,3,4-thia­diazol-2-amine)
Source: Acta Crystallogr E Crystallogr Commun. 2026 Jun 5;82(Pt 7):799–802. doi: 10.1107/S2056989026005517 (PMC13330805; doi:10.1107/S2056989026005517)
Supplement: Supplementary file 3 [file e-82-00799-sup3.pdf]

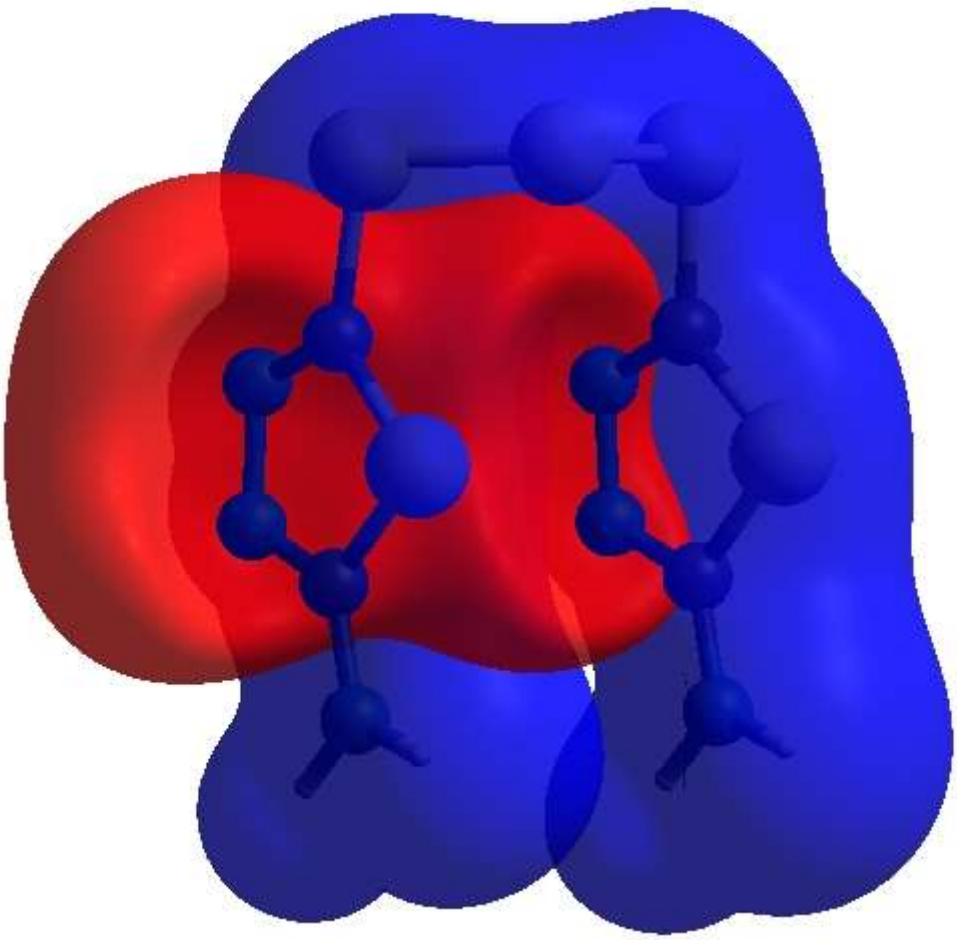

Figure S1. View of the three-dimensional Hirshfeld surface of the title compound plotted over electrostatic potential in the range of -0.0500 to 0.0500 a.u. using the STO-3 G basis set at the Hartree-Fock level of theory. Hydrogen-bond donors and acceptors are shown as blue and red regions around the atoms, corresponding to positive and negative potentials, respectively.
